# Solid malignant retroperitoneal masses—a pictorial review

**DOI:** 10.1007/s13244-013-0294-0

**Published:** 2013-11-29

**Authors:** Cressida Brennan, Dilkash Kajal, Korosh Khalili, Sangeet Ghai

**Affiliations:** 1 Division of Abdominal Imaging, Joint Department of Medical Imaging, University Health Network - Mount Sinai Hospital - Women’s College Hospital, University of Toronto, Toronto, ON Canada; 2 Primary Office, Toronto General Hospital, New Clinical Services Building, 1st Floor Rm 1C544, 585 University Avenue, Toronto, Ontario Canada M5G 2N2

**Keywords:** Retroperitoneal masses, Retroperitoneal neoplasms, Retroperitoneal space, Imaging

## Abstract

Primary retroperitoneal masses are a rare but important group of neoplasms. Cross-sectional imaging has revolutionised the investigation of patients with retroperitoneal neoplasms. Both computed tomography (CT) and magnetic resonance imaging (MRI) can contribute to tumour diagnosis, though histological confirmation is often required because of the considerable overlap of imaging features. Cross-sectional imaging is key to the pre-operative staging and planning of retroperitoneal masses, though ultrasound may also help in certain instances. Imaging also helps to select and guide the site to biopsy from these usually large and heterogeneous neoplasms. This article aims to review many of the primary retroperitoneal neoplasms that may be encountered by the radiologist.

## Introduction

The retroperitoneum in the abdomen is the space between the posterior parietal peritoneum anteriorly and the transversalis fascia posteriorly. It extends from the diaphragm superiorly to continue into the extraperitoneal space in the pelvis inferiorly. The retroperitoneum is loosely divided into the anterior and posterior pararenal, perirenal and great vessel spaces. The anterior pararenal space is bordered between the posterior parietal peritoneum anteriorly, the anterior renal or Gerota fascia posteriorly and laterally by the lateroconal fascia. This space includes the pancreaticoduodenal space and the pericolonic space. The posterior pararenal space lies between the posterior renal fascia and the transversalis fascia, whereas the perirenal space is located between the anterior and the posterior renal fascia. The great vessel space surrounds the aorta and the inferior vena cava (IVC) and is anterior to the vertebral bodies and psoas muscles. The anterior and posterior pararenal spaces merge inferior to the level of the kidneys, which communicates inferiorly with the prevesical space and extraperitoneal compartments of the pelvis [1].

The pelvic extraperitoneal space includes three intercommunicating potential extraperitoneal spaces: the prevesical, perivesical and perirectal spaces. The prevesical space is bordered by the infraumbilical abdominal wall anteriorly and the umbilicovesical fascia posteriorly, this space is also referred to as the space of Retzius. The perivesical space is below the pelvic peritoneal reflection, and contains the urinary bladder and the uterus (in females). The perirectal space surrounds the rectum and includes the peripheral presacral space [2].

Primary retroperitoneal neoplasms are a rare but important group of neoplasms. They account for only 0.1–0.2 % of all malignancies and arise outside the retroperitoneal organs [3]. Most primary retroperitoneal neoplasms arise from the mesodermal system with liposarcoma, leiomyosarcoma and malignant fibrous histiocytoma together, accounting for greater than 80 % of primary retroperitoneal sarcomas. The remaining primary retroperitoneal masses arise predominantly from the nervous system [1].

Owing to the loose connective tissue of the retroperitoneum, these masses tend to be large at the time of presentation [1]. They can be identified incidentally or may present clinically with a palpable abdominal or pelvic mass. Cross-sectional imaging has revolutionised the investigation of patients with retroperitoneal neoplasms. Both CT and MRI play an integral role in the characterisation of these masses and in evaluation of their extent and involvement of adjacent structures, and therefore in treatment planning. Whilst many authors have described useful imaging features to distinguish between the different entities [4], histological confirmation is required for diagnosis in most tumours because of overlap of imaging features and for tumour grading.

Both computed tomography (CT) and magnetic resonance imaging (MRI) can contribute to tumour characterisation, in determining the size of the lesion, its tissue content and relationship to adjacent organs and vessels. CT provides superior spatial resolution and detection of calcification, while MRI has superior soft tissue contrast and capabilities in the detection of fat within a lesion. Whilst there does remain significant overlap in the imaging characteristics of retroperitoneal neoplasms, some lesions have distinctive characteristics and can be diagnosed with some accuracy on imaging, e.g. aggressive angiomyxoma and liposarcoma (Table 1). Imaging also plays an important role in enabling histological diagnosis of these lesions. Retroperitoneal biopsies can be safely performed under CT or US guidance. Cross-sectional imaging with contrast is often able to recognise solid, vascular and the most dedifferentiated areas within these large heterogeneous masses, thereby enabling specific targeting within the mass.

**Table 1:** Key features of some of the primary retroperitoneal masses

Tumour	Characteristic feature
Liposarcoma	Macroscopic fat seen in CT or MR. Myxoid variety may have a pseudocystic appearance. Round cell or pleomorphic subtype may be predominantly soft tissue density.
Leiomyosarcoma	Large heterogeneous solid mass in the retroperitoneum and contiguous involvement of a vessel, is highly suggestive of a leiomyosarcoma.
Solitary fibrous tumour	Well defined masses with intense heterogeneous enhancement in the arterial phase that persists in the delayed phase. On MRI, multiple flow voids representing vascular channels may be identified on T2-weighted images.
Presacral myelolipoma	Seen in elderly female patients. Well-defined margins but indistinguishable from liposarcoma on imaging. Sulphur colloid scan may show uptake in the myeloid component.
Sacrococcygeal teratoma	Seen in children or young patients, more common in females. Variable appearance from cystic to mixed to solid. Bony defects may be present. Intralesional fat or calcification may be present.
Castleman disease	Intensely enhancing mass, with satellite nodules/nodes. Central low T1 and T2 signal on MRI may correspond to fibrosis.
Aggressive angiomyxoma	Young females. High T2 signal on MRI with characteristic whorled appearance and whorled appearance post contrast administration

The aim of this article is to illustrate many of the primary retroperitoneal masses and to review the associated imaging features.

## Liposarcoma

Liposarcoma is the most common sarcoma to occur in the retroperitoneum [3]. Retroperitoneal liposarcomas account for 10–15 % of all liposarcomas and about 30 % of retroperitoneal sarcomas [3]. They generally tend to occur in the 5th and 6th decades of life and can be classified into four subtypes: well differentiated with and without dedifferentiated components, myxoid and round cell liposarcoma and pleomorphic liposarcoma [1]. Different histological subtypes may co-exist in the same lesion [5]. Such histological subtypes are usually classified on the basis of the most aggressive cellular component.

**Fig. 1:**
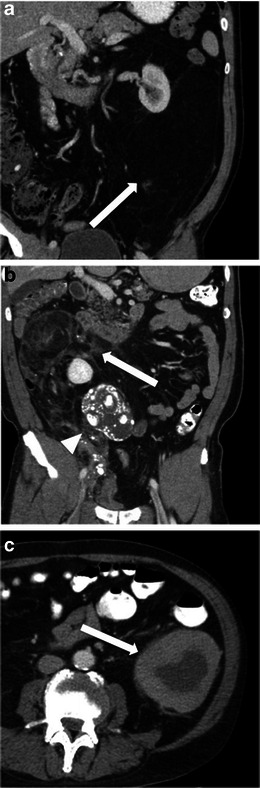
Imaging of liposarcoma **a** A 62-year-old woman with biopsy-proved well-differentiated liposarcoma. Note the large left retroperitoneal mass, which is of predominantly fatty attenuation (*arrow*). **b** A 56-year-old man with dedifferentiated liposarcoma. Coronal contrast-enhanced CT shows a large fatty right retroperitoneal mass with areas of soft tissue density (*arrow*) and coarse calcification (*arrowhead*). **c** Pleomorphic liposarcoma in a 60-year-old man. Note the enhancing retroperitoneal soft tissue mass with central necrosis (*arrow*)

Well-differentiated liposarcomas are typically round or lobulated and may displace or surround local structures. They contain predominantly fat. The fat content may be in excess of 75 % and these lesions must be distinguished from other fat-containing neoplasms which may occur in the retroperitoneum, such as lipoma, adrenal myelolipoma, renal angiomyolipoma and teratoma [5]. The fatty component is of high signal on T1-weighted imaging and demonstrates loss of signal with fat suppression. They demonstrate fat attenuation at CT (Fig. 1a). They frequently have thick, nodular septa. These septa and solid nodular components typically demonstrate enhancement after contrast administration [6]. Imaging helps to identify and to guide biopsy from the enhancing or soft tissue component of the mass, which is more likely to be the more aggressive component of the mass.

Histological dedifferentiation may occur within a portion of the well-differentiated liposarcoma, resulting in a more aggressive lesion, which is more prone to metastasise and carries a worse prognosis [1]. These lesions appear heterogeneous at CT and MRI with lack of distinction between fat and solid components. Calcification may be seen in up to 30 % of these tumours on CT and suggests dedifferentiation [6] (Fig. 1b), usually corresponding to osseus metaplasia, although they may represent osteosarcomatous or chondrosarcomatous elements.

Myxoid liposarcomas tend to occur in younger patients. They are of intermediate-grade malignancy and are typically lobular lesions with attenuation less than that of muscle on CT. The homogeneous dispersal of fat and soft tissue throughout the lesion gives a ‘pseudocystic’ appearance, near fluid density, on non-contrast enhanced CT. They characteristically show gradual, heterogeneous enhancement. Calcification can occasionally be seen. Pleomorphic/round cell liposarcomas are high-grade sarcomas. They are the least common subtype overall. They appear as heterogeneous soft-tissue masses with little or no fat within and may contain areas of necrosis (Fig 1c).

## Leiomyosarcoma

Leiomyosarcoma is the second most common primary retroperitoneal tumour in adults [3]. Leiomyosarcomas can be divided into three main categories: extravascular (62 %), intravascular (5 %) and a combination of both (33 %) [7]. They are believed to arise from blood vessels, spermatic cord or wolfian and mullerian duct remnants within the retroperitoneum [3].

**Fig. 2:**
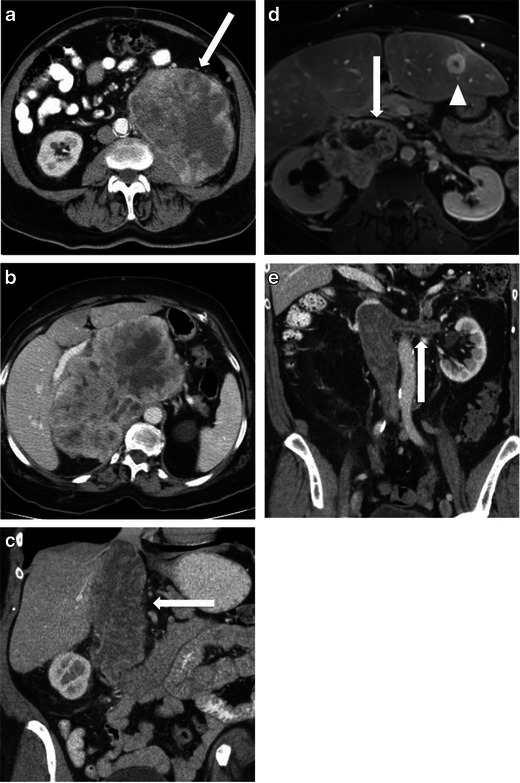
Imaging of leiomyosarcoma. **a** An 85-year-old man who presented with abdominal pain. CT shows a large heterogeneous left sided retroperitoneal mass (*arrow*) with central low attenuation in keeping with necrosis. **b** A 64-year-old woman presenting with early satiety. CT shows a large heterogeneous in the right abdomen, which is displacing the overlying liver. **c**, **d** A 60-year-old woman with IVC leiomyosarcoma. Coronal contrast-enhanced CT image (**c**) shows a large mass within the IVC (*arrow*). Axial T1-weighted image post gadolinium in the same patient (**d**) shows a heterogeneous enhancing mass centred on the IVC (*arrow*) and extending beyond the IVC into the paracaval location. There is moderate degree right-sided hydronephrosis secondary to the mass. Liver metastasis is noted in segment III of the liver (*arrowhead*). **e** A 77-year-old woman with IVC leiomyosarcoma extending to *left* renal vein. Coronal CT shows a heterogeneous enhancing mass filling the IVC and extending into the left renal vein (*arrow*)

At CT, these masses typically appear as large masses, often in excess of 10 cm. They have attenuation equal to or less to that of muscle and often have internal regions of low attenuation corresponding to areas of necrosis [7] (Fig. 2a, b). Areas of haemorrhage may occasionally be seen within. Tumours involving blood vessels such as the IVC may show polypoid intra-luminal extension with expansion of the blood vessel (Fig. 2c, d, e). The tumour thrombus typically enhances, in contrast to benign bland thrombus [1]. The presence of a heterogeneous large solid mass in the retroperitoneum with necrotic areas and contiguous involvement of a vessel, is highly suggestive of a leiomyosarcoma [1].

## Undifferentiated pleomorphic sarcoma

Undifferentiated pleomorphic sarcoma (UPS), previously called malignant fibrous histiocytoma (MFH), is the third most common retroperitoneal sarcoma [1]. It is the most common soft tissue sarcoma in late adult life, occurring primarily in the 50– to 60-year-old age group. Men are more commonly affected [1, 3].

**Fig. 3:**
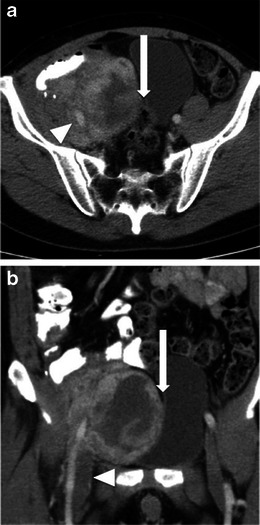
Imaging of MFH. A 57-year-old man who presented with abdominal pain. **a** Axial contrast enhanced CT shows a large right-sided heterogeneous mass with central low attenuation in keeping with necrosis. The mass is displacing local structures, including bladder and bowel loops (*arrow*). The right external iliac artery can be seen coursing through the mass (*arrowhead*). **b** Coronal reconstruction shows the extent of the mass and displacement of the bladder (*arrow*). There is thrombus within the right common femoral vein (*arrowhead*)

CT and MR imaging appearances are largely non-specific. Both modalities typically show a large, heterogeneously enhancing soft-tissue mass [8]. Areas of necrosis may be seen within, although these do not tend to be as extensive as those seen with leiomyosarcomas [3] (Fig. 3). CT may demonstrate internal calcification, which is typically peripheral and lumpy or ring-like on CT [9].

## Solitary fibrous tumour

Solitary fibrous tumours (SFTs) are a rare group of neoplasms, which arise from the mesenchyme and account for less than 2 % of all soft-tissue tumours [10]. Originally thought to exclusively arise from the pleura, it is now recognised that SFTs can arise from any site of the body and extrapleural SFTs are now thought to be more common. Most haemangiopericytomas are now classified as SFTs according to the World Health Organisation classification of soft-tissue tumours.

**Fig. 4:**
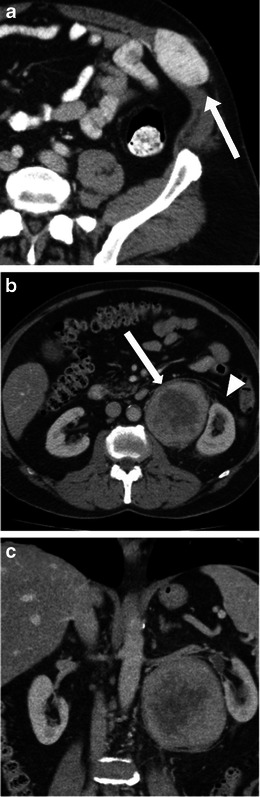
Imaging of solitary fibrous tumour (SFT). **a** A 40-year-old woman with a palpable abdominal wall. CT showed an intensely enhancing mass (*arrow*). SFTs in certain locations, such as the abdominal wall and the liver, are predominantly seen in female patients. **b**, **c** A 63-year-old man with a long-standing history of back pain. MRI of the spine incidentally picked up a mass in the retroperitoneum and CT of the abdomen was performed. Axial (**b**) contrast-enhanced CT shows a left sided large heterogeneous retroperitoneal mass (*arrow*) showing enhancement with central necrotic area, which is displacing and compressing the adjacent kidney causing mild hydronephrosis (*arrowhead*). Coronal CT (**c**) in the same patient shows the large mass causing distortion of the left kidney and left renal vessels

SFTs of the retroperitoneum are even more rare and only about 50 cases have previously been described in the literature [11]. Clinical presentation typically relates to the mass itself, i.e. palpable abdominal mass, pressure symptoms or may be detected incidentally. Up to 5 % of patients may present with hypoglycaemia, a symptom that is caused by excessive production of insulin growth factor 2 by the tumour itself [11]. They are usually benign, slow growing masses, though malignant SFTs are known to occur de novo or dedifferentiate from benign SFTs. Extra-pleural SFTs in certain locations, such as the abdominal wall and liver have been found predominantly in female patients (Fig. 4a).

At imaging, SFTs usually appear as well-defined masses with intense heterogeneous enhancement in the arterial phase, indicating its hypervascular nature, which persists in the delayed phase because of the fibrous component in the mass. Areas of necrosis, haemorrhage or cystic changes may be seen, though calcification is rare (Fig. 4b, c). On MRI, multiple flow voids representing vascular channels may be identified on T2-weighted images [2].

## Dermatofibrosarcoma protuberans

**Fig. 5:**
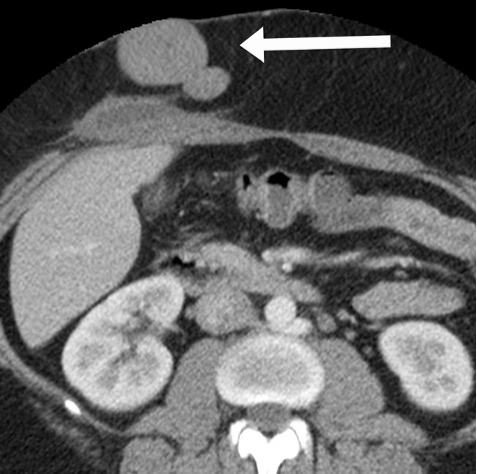
Imaging of dermatofibrosarcoma protuberans. A 29-year-old woman who presented with an abdominal wall mass in a transverse incision. CT at the time of presentation shows soft-tissue masses in the right anterior abdominal wall (*arrow*). These were resected and histopathology showed DFSP. These masses typically involve the skin on imaging (dermal layer) and show moderate soft tissue enhancement

Dermatofibrosarcoma protuberans (DFSP) is a rare soft tissue tumour, which tends to occur at a young age, generally in the 1st–5th decades. This tumour occurs slightly more commonly in male patients. It arises from the dermal layer of the skin and is classified as a sarcoma. These tumours typically arise on the trunk, head and neck region and on the extremities [12]. It can on occasion, metastasise to unusual sites, including the retroperitoneum [13] (Fig. 5). Fibrosarcomatous transformation or transformation to malignant fibrous histiocytoma is a rare but well-recognised entity.

CT appearances that have been described include a well-defined lesion with tissue attenuation equal to or slightly higher than that of skeletal muscle. Moderate enhancement may be seen post intravenous contrast administration [12].

## Synovial sarcoma

**Fig. 6:**
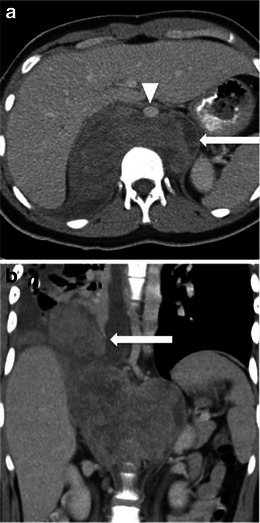
Imaging of synovial sarcoma. An 18-year-old man who presented with shortness of breath. **a** Axial contrast-enhanced CT shows a large heterogeneous retroperitoneal/retrocrural soft-tissue mass (*arrow*), which is displacing the aorta (*arrowhead*). **b** Coronal CT shows the extension of the retroperitoneal mass into the mediastinum. There is a right-sided pleural effusion and soft tissue pleural metastases (*arrow*)

Synovial sarcoma is extremely rare in the retroperitoneum. In the retroperitoneum, it is associated with an extremely poor prognosis. They typically occur in younger patients, aged 15–40. Imaging findings are non-specific and the diagnosis should be considered in a young patient presenting with a mass in the retroperitoneum [14] (Fig. 6).

At CT, these lesions tend to be hypoattenuating, with peripheral irregular enhancement and central necrosis. Calcification may also be seen. On MR imaging, the heterogeneous mass is typically isointense on T1-weighted imaging and hyperintense on T2-weighted imaging [1, 14].

### Extra-adrenal myelolipoma

**Fig. 7:**
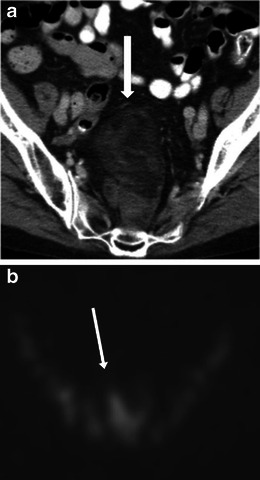
Imaging of myelolipoma. An 80-year-old woman with an incidental pre-sacral mass identified on CT (**a**). The mass (*arrow*) is heterogeneous with predominant fat attenuation and some solid enhancing components. The appearances are similar to a liposarcoma. Biopsy revealed mature adipose tissue and myeloid contents. A sulphur colloid scan (**b**) shows uptake in the pre-sacral region (*arrow*) confirming the diagnosis of extra-adrenal myelolipoma

Myelolipoma is a benign tumour that consists of mature adipose tissue and normal trilineage hematopoietic components. About 15 % arise outside the adrenal gland and the pre-sacral location accounts for 50 % of the extra-adrenal myelolipomas [15]. Other extra-adrenal locations include perirenal, retroperitoneum, mediastinum, liver and stomach [16]. The presarcral myelolipomas occur predominantly in elderly women, are usually asymptomatic, though may present with pressure symptoms on rectum, ureters or urinary bladder. There has also been mention of their association with Cushing syndrome, Addision disease and exogenous steroid use [17]. On imaging, they are seen as predominantly fat-containing masses with areas of soft-tissue attenuation and are indistinguishable from liposarcomas (Fig. 7a). Sulphur colloid scan may show uptake in the myeloid components of the mass (Fig. 7b). Unlike teratomas, they are seen in the older population [2].

### Sacrococcygeal teratoma

Teratoma is a germ cell tumour with elements from more than one germ cell layer. They are commonly seen in infants and children, and tumours in adults are more likely to be malignant. In adults, they are about 4 times more commonly seen in women [18, 19].

**Fig. 8:**
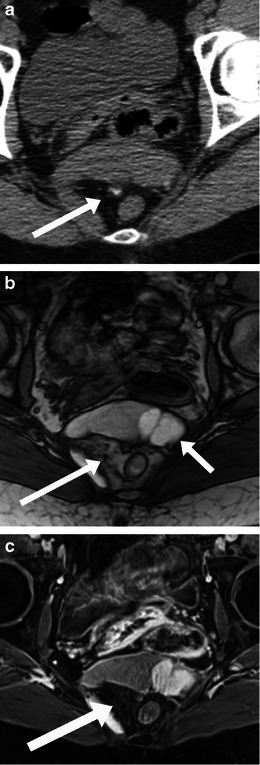
Imaging of sacrococcygeal teratoma. A 49-year-old woman with benign cystic teratoma. **a** Unenhanced axial CT scan performed for renal colic showed a heterogeneous presacral mass with areas of soft-tissue density, fat density and a speck of calcification (*arrow*). Subsequent MRI shows areas of fat which show signal drop (*long arrows*) on the T1-weighted fat saturated images (**c**), and areas of haemorrhage (*short arrows*, **b**) which do not show signal drop

Clinically, they may be asymptomatic or present with mass or pressure symptoms [2]. On imaging, they can have a variable appearance from solid to cystic or mixed areas of solid and cystic component. Intralesional fat and calcification may be present (Fig. 8). The sacral or coccyx bone may be involved.

## Aggressive angiomyxoma

**Fig. 9:**
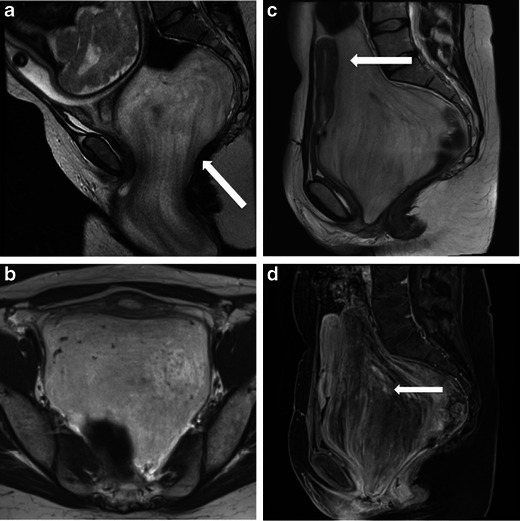
Imaging of aggressive angiomyxoma. **a** A 38-year-old woman presented with abdominal pain during pregnancy. Sagittal T2-weighted MRI image shows a large pelvic mass (*arrow*), which is predominantly T2 hyperintense with a ‘whorled’ appearance. **b**, **c** Follow-up imaging post-partum showed interval growth of the mass. The mass is displacing structures, the uterus can be seen displaced anteriorly (*arrow*). **d** Sagittal T1-weighted image post gadolinium shows the characteristic swirling pattern of contrast enhancement, typical of aggressive angiomyxoma (*arrow*)

Aggressive angiomyxoma is an uncommon benign mesenchymal tumour. It predominantly involves the pelvis and perineum of women of childbearing age, typically between the 2nd and 4th decades [20]. Most tumours are large at diagnosis and grow slowly. Clinical presentation may include discomfort from the mass, visible mass or pressure effects on adjacent pelvic organs [21]. It tends to displace structures such as urethra, vagina and rectum, rather than invade them (Fig. 9a–c), though on pathology they are seen as poorly circumscribed.

These are one of the few primary extraperitoneal masses which have a distinctive and characteristic appearance on MR imaging. On CT, these tumours are typically of low attenuation relative to muscle. On MRI, these lesions are isointense to muscle on T1-weighted imaging, and hyperintense on T2-weighted imaging (from the high myxomatous component in these tumours) with a distinctive whorled appearance [20, 21]. Following contrast enhancement, they show a characteristic swirled, layered or ‘onion-peel’ appearance (Fig. 9d). MRI also helps in evaluation of tumour extent and thereby in surgical planning. Extension across the pelvic diaphragm can be easily identified on MRI and helps plan the surgical approach. High local recurrence rates are usually related to incomplete excision. Incomplete excision may be secondary to incorrect assessment of actual extent of tumour on preoperative imaging or because of the proximity of the mass to important structures such as the urethra, vagina or the sphincters [22].

### Castleman disease

Castleman disease, a benign giant lymph node hyperplasia, is an uncommon benign lymphoproliferative disorder. Castleman disease may occur anywhere along the lymphatic chain, and about 70 % occur in the mediastinum. Extrathoracic sites include neck, axilla, mesentery, pelvis, pancreas, adrenal and retroperitoneum. A slight female predominance is reported in the literature. In the retroperitoneum, they are more common on the left side.

There are two major histological types: the hyaline-vascular type, and the plasma cell type. Clinically, the hyaline-vascular type is usually benign, localised and asymptomatic, whereas the less common plasma cell variant may be disseminated, malignant with systemic symptoms such as fever, anaemia, weight loss and night sweats [23].

**Fig. 10:**
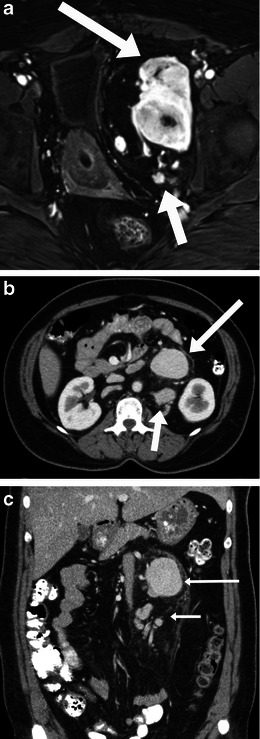
Imaging of Castleman disease. **a** Localised Castleman disease in a 48-year-old woman. Axial T1 weighted post gadolinum subtracted MRI image shows an intensely enhancing dominant mass (*long arrow*) with satellite nodules (*short arrow*) in the left extraperitoneal location in the pelvis. Axial (**b**) and coronal (**c**) contrast-enhanced CT scan images show an enhancing dominant mass (*long arrow*) with multiple smaller satellite nodules (*short arrow*) in the left retroperitoneum in a 36-year-old woman with localised Castleman disease

The characteristic imaging features of localised and hyaline vascular type of Castleman disease include a well-defined solitary mass or a dominant mass with small satellite nodules, showing intense enhancement (Fig. 10). Central areas of fibrosis, if seen, is one of the characteristic features, and manifests as hypointense signal on both T1- and T2-weighted images [24]. Calcification may be seen in a small number. Central cystic areas or necrosis is rare. Main differentials include metastatic disease from a primary such as renal cell carcinoma and melanoma, but the presence of a dominant hyperenhancing mass with satellite nodules localised to one area is characteristic for Castleman disease.

The plasma cell or disseminated form of Castleman disease may show hepatosplenomegaly, ascites and lymphadenopathy, which generally shows less enhancement than in the localised form [23].

### Lymphangioma and lymphangiomatosis

Lymphangiomas are congenital benign vascular lesions that result as a result of sequestration of embryonic lymphatic vessels that fail to communicate with the rest of the lymphatic or venous channels. They occur more commonly in children, though adult clinical presentation is not rare. Less than 1 % of lymphangiomas occur in the retroperitoneum [4].

**Fig. 11:**
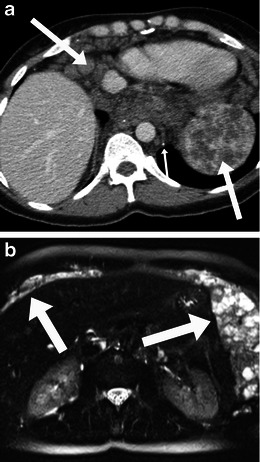
Imaging of lymphangiomatosis. **a** Axial CT image in a 38-year-old woman shows a low density mass (*arrows*) with specks of calcification (*small arrow*) scattered across the fascial planes in the retroperitoneum, spleen, epoicardial and retrocrurual spaces. Spleen is also involved. The patient was asymptomatic and the scan had been performed for a lump/desmoid tumour in the rectus sheath. **b** Axial T2-weighted MRI image shows the mass to be multicystic in nature (*arrows*) with septations

Lymphangiomatosis is a rare disease with multifocal sites of lymphatic proliferation that are locally infiltrative, with growth along tissue planes [25, 26]. It typically presents during childhood and may involve multiple parenchymal organs including the lung, liver, spleen, bone, and skin. On imaging they appear as well-defined multicystic masses with enhancement in the septations (Fig. 11). Fine specks of calcification may be seen [1].

## Neurogenic tumours

Neurogenic tumours constitute up to 20 % of the primary retroperitoneal masses [1]. Majority of neurogenic tumours are benign and are seen in younger population. Neurogenic tumours are classified according to their cells of origin [27] (Table 2). Schwannomas and neurofibromas arise from the nerve sheath; ganglioneuromas, ganglioneuroblastomas and neuroblastomas from sympathetic nerves; and paragangliomas and pheochromocytomas from chromaffin cells. Neuroblastoma and ganglioneuroblastoma are malignancies of childhood and are very rare in adults.

**Table 2:** Classification of neurogenic tumours

Cell of origin	Benign	Malignant
Nerve Sheath origin	Schwannoma, neurofibroma and neurofibromatosis Neurilemmomas	Neurogenic sarcoma/malignant schwannoma
Sympathetic nerves/ganglionic origin	Ganglioneuroma	Ganglioneuroblastoma, neuroblastomas
Chromaffin/paraganglionic cells	Pheochromocytoma, paraganglioma	Both paraganglioma and pheochromocytoma can be malignant; cortical carcinoma

## Neurofibromas and nuerofibromatosis

Neurofibromas are seen more commonly in the 2nd–4th decades, predominantly as isolated sporadic tumours, and are more common in men. In approximately 10 %, neurofibromas may be associated with neurofibromatosis (NF) 1 and 2. NF1 is commonly an autosomal dominant genetic disorder due to mutation of chromosome 17. In up to 50 % of patients, it can be a sporadic mutation. It comprises of hamartomas, hyperplasia and neoplastic lesions of neuroectodermal or mesenchymal origin. There are many clinical manifestations such as neurofibromas, café au lait spots, Lisch nodules and axillary/inguinal freckling. Multiple plexiform neurofibromas, cutaneous neurofibromas and Lisch nodules are specific to NF1 [28].

**Fig. 12:**
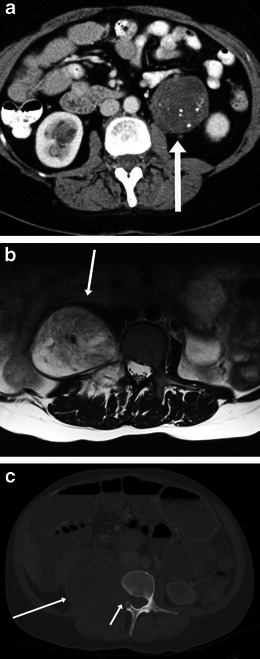
Imaging of neurofibroma. **a** CT scan in a 64-year-old woman showed an incidental well defined hypodense mass with speck of calcification (*arrow*) in the left retroperitoneum. **b**, **c** A 45-year-old woman with lower abdomen/thigh pain. Axial T2 MRI (**b**) showing a heterogeneous, high signal lobulated mass at L3/L4 level (*arrow*). The right L3 nerve root was not separately identified. CT scan (bone window settings) at the same level (**c**) shows the mass (*arrow*) exiting from the widened neural foramina without causing bony destruction (*small arrow*)

On CT, retroperitoneal neurofibromas are well-defined, round, hypodense, inhomogeneous solid masses, which may be seen along the expected course of the nerve. Widening of the neural foramina may be present without bony destruction (Fig. 12). These are usually well defined and demonstrate low-level uniform enhancement. Multiple cystic areas may also be seen following contrast enhancement. On MR, neurofibromas are low T1 and high T2 signal masses. The fibrous tissue within the neurofibromas is usually low on T2, sometimes giving rise to a target appearance. The fibrous core usually enhances after gadolinium administration in a whorled pattern.

**Fig. 13:**
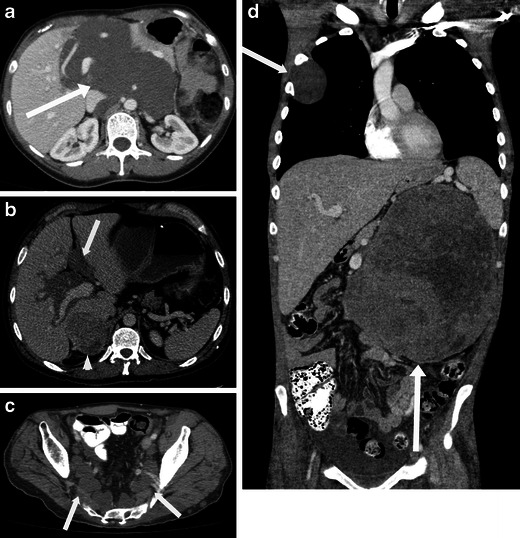
Imaging in neurofibromatosis (NF-1). **a** Plexiform neurofibromatosis in a 36-year-old woman with NF-1. Axial contrast enhanced image from upper abdomen shows a large lobulated hypodense mass (*arrow*) encasing the vessels without causing any narrowing. The adjacent organs are displaced by the central mass without invasion. **b** Plexiform neurofibromatosis and pheochromocytoma in a 45-year-old man with NF-1. Contrast enhanced CT scan shows an ill-defined hypodense plexiform neurofibromatosis mass (*arrow*) extending along the portal triad in the liver. The heterogeneously enhancing right adrenal mass (*arrowhead*) was a pheochromocytoma on resection. **c**, **d** A 29-year-old man with NF-1. Multiple low density well-defined hypodense neurofibromas along the sacral plexus were seen in the pelvis on both sides (*arrows*) on CT scan (**c**). On a follow up CT scan (**d**), a large new heterogeneous solid mass (*arrow*) was seen in left retroperitnoeum, proven to be a malignant peripheral nerve sheath tumour on resection. A neurofibroma is also seen in the right chest (*arrow*)

Plexiform neurofibromas are seen almost exclusively in NF 1. They are hypodense on CT from presence of adipose tissue, myxoid tissue and schwann cells (Fig. 13). They tend to be large and lumbosacral plexus is the commonest site in the retroperitoneum [29].

There is up to 5 % lifetime risk of malignant transformation of neurofibroma to malignant peripheral nerve sheath tumour (MPNST)—previously known as neurofibrosarcoma (Fig. 13d) [30]. These are highly aggressive and infiltrative tumours, may present with distant metastases and demonstrate high recurrence rate. The most common location of MPNST in retroperitoneum is in the paraspinal region. They may be clinically asymptomatic and imaging is not reliable in differentiating between benign and malignant tumours. The helpful CT/MR features include inhomogeneity with necrotic areas, irregular/infiltrative borders with invasion of surrounding structures and destruction of adjacent bones [27].

## Schwannoma (neurolemmoma)

Schwannomas are the most common retroperitoneal neurogenic tumours, comprising around 5 % of all retroperitoneal tumours. They are benign tumours and arise from the nerve sheaths of peripheral nerves and are most commonly located in the paravertebral region. Schwannomas are seen in young adults, but unlike neurofibromas, they are twice as common in women [27].

**Fig. 14:**
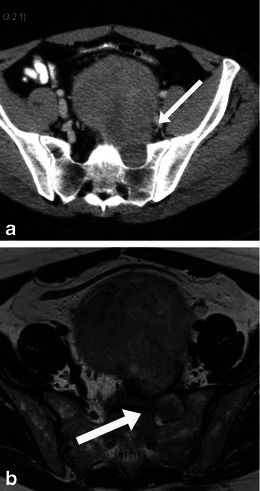
Imaging of cellular schwannoma. Axial contrast-enhanced CT scan (**a**) and axial T2-weighted MRI scan (**b**) show a heterogeneously enhancing soft-tissue mass (*arrows*) in a 26-year-old woman. The left S2 neural foramina is widened with bony destruction

**Fig. 15:**
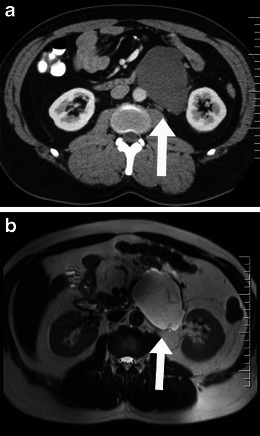
Imaging of cystic schwannoma. Cystic schwannoma in a 46-year-old man. The mass was detected incidentally. Axial enhanced CT scan (**a**) showed a well defined hypodense mass (*arrow*) in the left retroperitoneum. On axial T2-weighted MRI image (**b**) the mass (*arrow*) was seen to be predominantly cystic with septations

Histologically, they consist of two different components: Antoni type A areas are highly cellular (Fig. 14), while Antoni type B areas are cystic and oedematous (Fig. 15). On imaging, they tend to be indistinguishable from neurofibromas, but central degenerative changes (necrosis, haemorrhage, calcification) may be seen in schwannomas (ancient schwannoma).

### Ganglioneuromas

Ganglioneuromas occur in adolescents and young children. They are rare, benign neurogenic tumours from the sympathetic ganglia and may arise anywhere along the paravertebral sympathetic plexus or occasionally from the adrenal medulla. They are often detected incidentally even if large, or may present with pain or mass on deep palpation. Occasionally they may secrete catecholamines, vasoactive intestinal polypeptide, or androgenic hormones [27].

**Fig. 16:**
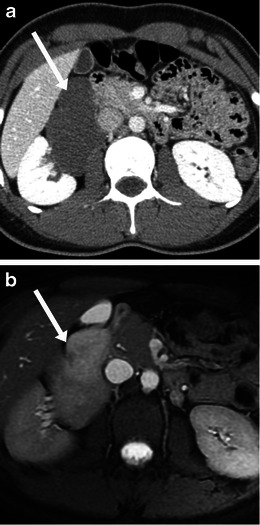
Imaging of ganglioneuroma. An 18-year-old woman with incidentally detected mass on ultrasound at age of 12. Subsequent biopsy showed ganglioneuroma on histology. **a** Contrast-enhanced CT scan shows a well-defined lobulated hypodense mass (*arrow*) in right retroperitoneum without invading any of the adjacent structures. **b** Axial T2-weighted MRI image shows hyperintense signal from the myxoid stroma (*arrow*). **c** Axial T1-weighted image post gadolinium shows mild gradual enhancement (*arrow*)

On imaging, they appear as well-defined lobulated masses. Discrete or punctate calcification may be seen in up to 20 %. Gradual enhancement is usually seen post contrast (Fig. 16). On MRI, they usually have a high T2 signal from the myxoid stroma within them with linear bands of low signal from schwann cells or collagen [31–33].

## Paraganglioma

Paragangliomas constitute 10 % of all pheochromocytomas arising from the neural crest cells along the sympathetic chain. The organ of Zuckerkandl located along the aorta just superior to the bifurcation is the most common location. The trigone of the urinary bladder usually contains cells of neural crest origin and, therefore, a rare possible site. A significant number produce high catecholamine levels, causing symptoms such as palpitations, tachycardia, flushing, sweating, etc.

Paragangliomas may be associated with von Hippel Lindau syndrome, MEN Type II and NF1, and are generally more malignant with higher metastatic rate than their adrenal counterparts (up to 40 % of parangliomas compared with up to 10 % of pheochromocytomas) [34].

**Fig. 17:**
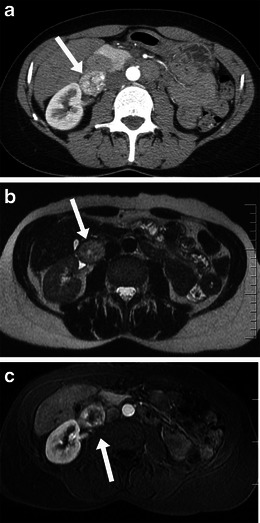
Imaging of paraganglioma. A 43-year-old woman with family history of paraganglioma. Her 24-h urine normetanephrine and norepinephrine levels were elevated. **a** A well-defined enhancing mass with central necrosis (*arrow*) was seen in the right retroperitoneum. The right adrenal was normal. Axial T2-weighted image (**b**) shows hyperintense signal (*arrow*), while post gadolinium T1-weighted MRI image (**c**) shows intense enhancement (*arrow*)

On CT, these are large, lobulated and heterogeneous masses due to presence of necrosis, blood and calcification and are mostly hypervascular after contrast administration. On MRI, they are heterogeneously bright on T2-weighted imaging (Fig. 17), though less than 80 % of paragangliomas show the characteristic uniform high signal on T2-weighted imaging because of haemorrhage within them [35]. Haemorrhagic portions can be bright on T1-weighted sequences. Fluid-fluid levels can also be seen in some cases.

## Conclusion

Primary retroperitoneal neoplasms are rare. Cross-sectional imaging is key to the evaluation of retroperitoneal masses and in the pre-operative staging and surgical planning of these lesions. This article has reviewed the imaging findings of many of these masses that may be encountered by the radiologist in their practice.

## References

[1] Rajiah P, Sinha R, Cuevas C, Dubinsky TJ, Bush WH, Kolokythas O (2011). Imaging of uncommon retroperitoneal masses. Radiographics.

[2] Shanbhogue AK, Fasih N, Macdonald DB, Sheikh AM, Menias CO, Prasad SR (2012). Uncommon primary pelvic retroperitoneal masses in adults: a pattern-based imaging approach. Radiographics.

[3] Neville A, Herts BR (2004). CT characteristics of primary retroperitoneal neoplasms. Crit Rev Comput Tomogr.

[4] Nishino M, Hayakawa K, Minami M, Yamamoto A, Ueda H, Takasu K (2003). Primary retroperitoneal neoplasms: CT and MR imaging findings with anatomic and pathologic diagnostic clues. Radiographics.

[5] Song T, Shen J, Liang BL, Mai WW, Li Y, Guo HC (2007). Retroperitoneal liposarcoma: MR characteristics and pathological correlative analysis. Abdom Imaging.

[6] Craig WD, Fanburg-Smith JC, Henry LR, Guerrero R, Barton JH (2009). Fat-containing lesions of the retroperitoneum: radiologic-pathologic correlation. Radiographics.

[7] Hartman DS, Hayes WS, Choyke PL, Tibbetts GP (1992). Leiomyosarcoma of the retroperitoneum and inferior vena cava: radiologic-pathologic correlation. Radiographics.

[8] Paling MR, Hyams DM (1982). Computed tomography in malignant fibrous histiocytoma. J Comput Assist Tomogr.

[9] Ko SF, Wan YL, Lee TY (1998). CT features of calcifications in abdominal malignant fibrous histiocytoma. Clin Imaging.

[10] Gold JS, Antonescu CR, Hajdu C, Ferrone CR, Lewis JJ, Hussain M, Brennan MF, Coit DG (2002). Clinico-pathologic correlates of solitary fibrous tumours. Cancer.

[11] Shanbhogue AK, Prasad SR, Takahasi N, Vikram R, Zaheer A, Sandrasegaran K (2011). Somatic and visceral solitary fibrous tumors in the abdomen and pelvis: cross-sectional imaging spectrum. Radiographics.

[12] Schepper AM, Vanhoenacker FM, Parizel PM, Gielen JLMA (eds)(2006) Imaging of soft tissue tumors, 3rd edn. Springer, Berlin Heidelberg New York

[13] Eisen RN, Tallini G (1993). Metastatic dermatofibrosarcoma protuberans with fibrosarcomatous change in the absence of local recurrence. A case report of simultaneous occurrence with a malignant giant cell tumor of soft tissue parts. Cancer.

[14] Ulusan S, Kizilikilic O, Yildirim T, Hurcan C, Bal N, Nursal TZ (2005). Radiological findings of primary retroperitoneal synovial sarcoma. Br J Radiol.

[15] Dann PH, Krinsky GA, Israel GM (2008). Case 135: presacral myelolipoma. Radiology.

[16] Kammen BF, Elder DE, Fraker DL, Siegelman ES (1998). Extraadrenal myelolipoma: MR imaging findings. AJR Am J Roentgenol.

[17] Singla AK, Kechejian G, Lopez MJ (2003). Giant presacral myelolipoma. Am Surg.

[18] Bryant P, Leditschke JF, Hewett P (1996). Hereditary presacral teratoma. Aust N Z J Surg.

[19] Bull J, Yeh KA, McDonnell D, Caudell P, Davis J (1999). Mature presacral teratoma in an adult male: a case report. Am Surg.

[20] Stewart ST, McCarthy SM (2004). Case 77: aggressive angiomyxoma. Radiology.

[21] Sinha R, Verma R (2007). Case 106: aggressive angiomyxoma. Radiology.

[22] Brunelle S, Bertucci F, Chetaille B, Lelong B, Piana G, Sarran A (2013). Aggressive angiomyxoma with diffusion-weighted magnetic resonance imaging and dynamic contrast enhancement: a case report and review of the literature. Case Rep Oncol.

[23] Kim TJ, Han JK, Kim YH, Kim TK, Choi BI (2001) Castleman disease of the abdomen: imaging spectrum and clinicopathologic correlations. J Comput Assist Tomogr 25:207–214 10.1097/00004728-200103000-00008 11242214

[24] Zhou LP, Zhang B, Peng WJ, Yang WT, Guan YB, Zhou KR (2008). Imaging findings of Castleman disease of the abdomen and pelvis. Abdom Imaging.

[25] Wunderbaldinger P, Paya K, Partik B (2000). CT and MR imaging of generalized cystic lymphangiomatosis in pediatric patients. AJR Am J Roentgenol.

[26] Marom EM, Moran CA, Munden RF (2004). Generalized lymphangiomatosis. AJR Am J Roentgenol.

[27] Rha SE, Byun JY, Jung SE, Chun HJ, Lee HG, Lee JM (2003) Neurogenic tumors in the abdomen: tumor types and imaging characteristics. Radiographics 23:29–43 10.1148/rg.231025050 12533638

[28] Levy AD, Patel N, Dow N, Abbott, Miettinen M, Sobin LH (2005). Abdominal neoplasms in patients with neurofibromatosis type 1: radiologic-pathologic correlation. Radiographics.

[29] Bass JC, Korobkin M, Francis IR, Ellis JH, Cohan RH (1994). Retroperitoneal plexiform neurofibromas: CT findings. AJR Am J Roentgenol.

[30] Gutmann DH, Aylsworth A, Carey JC (1997). The diagnostic evaluation and multidisciplinary management of neurofibromatosis 1 and neurofibromatosis 2. JAMA.

[31] Radin R, David CL, Goldfarb H, Francis IR (1997). Adrenal and extra-adrenal retroperitoneal ganglioneuroma: imaging findings in 13 adults. Radiology.

[32] Otal P, Mezghani S, Hassissene S (2001). Imaging of retroperitoneal ganglioneuroma. Eur Radiol.

[33] Zhang Y, Nishimura H, Kato S (2001). MRI of ganglioneuroma: histologic correlation study. J Comput Assist Tomogr.

[34] Enzinger FM, Weiss SW (1988). Soft tissue tumors.

[35] Francis IR, Korobkin M (1996). Pheochromocytoma. Radiol Clin N Am.

